# Symptomatic Refluxing Distal Ureteral Stump after Retroperitoneoscopic Nephrectomy

**Published:** 2014-04-01

**Authors:** Marianna Iaquinto, Ciro Esposito, Maria Escolino, Alessandra Farina, Alessandro Settimi, Bruno Cigliano

**Affiliations:** Department of Pediatric Surgery “Federico II” University of Naples, Italy

A 9-year-old girl with grade 4-5 right vesico-ureteric reflux (VUR) into a solitary collecting system was initially treated with endoscopic Deflux injection. Micturition cystourethrogram (MCUG) performed after 6 months showed a grade 1-2 reflux. After 3 years, MCUG was repeated due to recurrent urinary tract infection (UTI) showed severe VUR. DMSA renal scan showed a non-functioning right kidney. The patient underwent a right nephrectomy by retroperitoneoscopic approach. A small distal ureteral stump (DUS) was left. One year after surgery, another MCUG was performed due to recurrent UTI showed an active VUR in the ureteral stump (Fig.1). The 5-cm long DUS (Fig. 2) was surgically removed. The child is asymptomatic at follow-up of 5 years.

**Figure:**
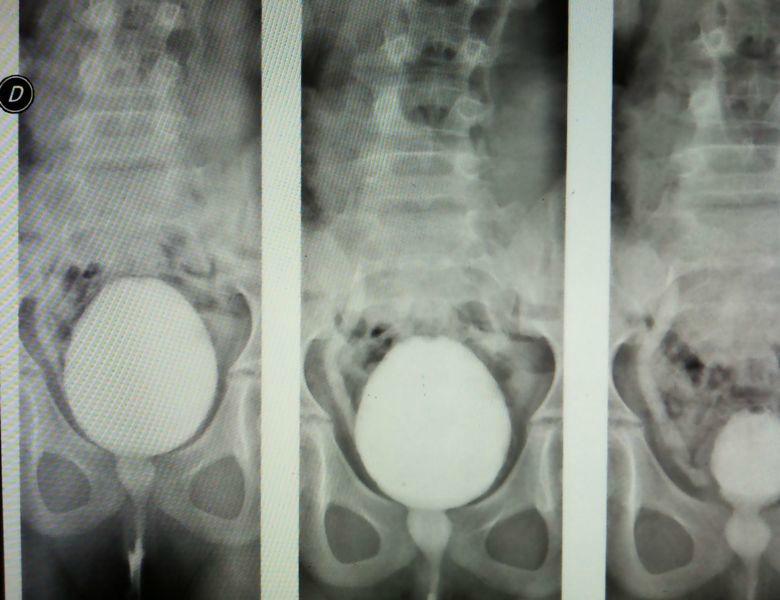
Figure 1: MCUG shows an active VUR in the ureteral stump.

**Figure:**
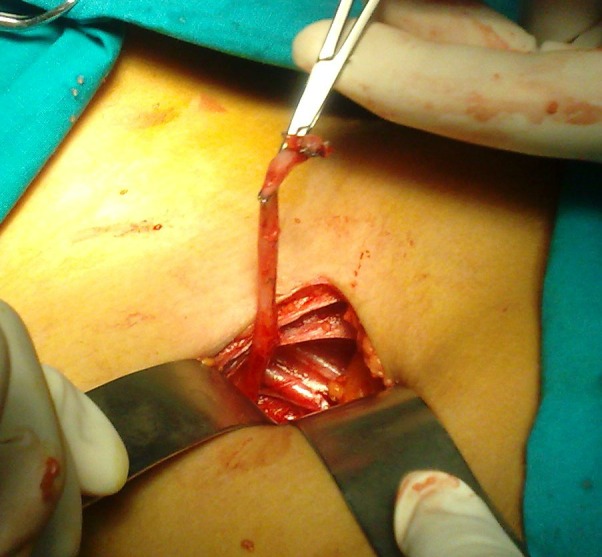
Figure 2: The ureteral stump.

## DISCUSSION

The management of a poorly functioning kidney associated with primary VUR is nephrectomy with total or proximal ureterectomy. The retroperitoneal laparoscopic approach is particularly beneficial owing to a small residual distal ureteral stump (DUS) shorter than that achievable via a single flank incision. However, it does not allow the stump dissection down to the bladder base.[1] The transperitoneal laparoscopic approach is preferable for total removal of the ureter.[2]

Reflux in DUS is a rare complication due to partial excision of ureter. Recurrent UTI after nephrectomy is a useful signal to suspect the presence of reflux in DUS which act as a reservoir resulting in stasis and infections.[4] A long stump could be a risk factor in the pathogenesis of ureteric stump syndrome. A long refluxing stump inevitably acts as a diverticulum from which the urine cannot be effectively drained thus leading to outbreaks of UTI.[3] In our case a short ureteral stump became symptomatic due to reflux into it. We recommend transperitoneal laparoscopic approach for the management of a poorly functioning kidney which allows nephrectomy and complete ureterectomy up till the bladder base thus avoids reflux into the DUS.

## Footnotes

**Source of Support:** Nil

**Conflict of Interest:** None declared

